# Highly Efficient Synthesis of Carbon-Based Molybdenum Phosphide Nanoparticles for Electrocatalytic Hydrogen Evolution

**DOI:** 10.1186/s11671-020-3246-x

**Published:** 2020-01-09

**Authors:** Yang Li, Lun Cai, Qilin Huang, Jun Liu, Ranran Tang, Wenhan Zhou

**Affiliations:** School of Mechanics and Optoelectronic Physics, Anhui University of Science and Technology, Huainan, 232001 People’s Republic of China

**Keywords:** Molybdenum phosphide, Electrocatalysis, Nanoparticles, Hydrogen evolution reaction

## Abstract

Molybdenum phosphide in transition metal phosphides members are considered as an attractive electrocatalyst for hydrogen evolution reaction (HER). However, its unsatisfactory stability and conductivity in an alkaline environment has dragged on its development. Here, we successfully introduced N, C co-doped MoP (MoP-NC) nanoparticles by a simple and efficient two-step synthesis method using urea as a carbon source into the molybdenum phosphide system. The cheapness of urea and the excellent carbon to nitrogen ratio remove the obstacles ahead of the development of MoP-NC composites. The obtained composites have excellent HER electrocatalytic activity and stability in 1-M potassium hydroxide (KOH) solution, which requires only an overpotential of 131 mV to achieve a current density of 10 mA cm^−2^ and exhibits negligible performance degradation after 1000 CV cycles.

## Background

In recent years, the rapid development of human beings has led to the gradual depletion of fossil energy [1–3]. Therefore, researchers are striving to find an environmentally friendly energy source to curb this vicious circle, so that hydrogen can be held on the throne of energy. However, traditional photolysis and electrolysis of water to produce hydrogen are in a difficult bottleneck in terms of efficiency. Whereas with the introduction of high-efficiency electrolyzed water-catalyzed hydrogen production catalysts, people have made a qualitative leap in the mass production of hydrogen. This catalyst-mediated electrocatalytic process requires the catalyst itself to have low hydrogen evolution reaction (HER) overpotential. Although the noble metals such as Pt at this stage have extremely low overpotential and excellent stability, they are expensive [4–6]. The scarcity of available soil limits the large-scale application of such catalysts [7–9]. Therefore, finding a material with low cost and relatively good electrocatalytic hydrogen evolution performance has been a hot spot in the past few years [10–13].

It is worth noting that recently researchers have found that some non-precious metal catalysts have a good price/performance ratio in the direction of hydrogen evolution, among which the most widely used Molybdenum phosphide (MoP) in transition metal phosphides (TMPs) [14–17]. Sun et al. mixed (NH_4_)_6_Mo_7_O_24_·4H_2_O, (NH_4_)_2_HPO_4_ and citric acid (CA) in different molar ratios, when Mo:P:CA = 1:1:*x* and *x* = 2, the formation of crosslinked network structure MoP nanoparticles have the best HER performance [18]. Joshua et al. prepared amorphous MoP nanoparticles with good HER properties by heating hexacarbonyl molybdenum and trioctylphosphine (TOP) [19]. However, the conductivity of pure molybdenum phosphide is not satisfactory, and the hydrogen evolution performance and stability in an alkaline solution are not as good as in an acidic environment, so that the conductivity and stability can be enhanced by introducing a carbon-based material [20–22].

We achieved high-efficiency two-step synthesis by introducing urea as a carbon source in the molybdenum phosphide system, and successfully prepared N, C co-doped MoP (MoP-NC) nanoparticles, which have excellent catalytic activity and stability even in alkaline electrolytes. In addition, we designed two control groups that explored urea action including no carbon source and glucose instead of urea as the carbon source. Interestingly, the former is always weaker than the latter when using glucose and urea as carbon sources, respectively. This can be attributed to the role of urea as both a carbon source and a nitrogen source for the auxiliary synthesis of molybdenum phosphide [23].

## Presentation of the Hypothesis

Molybdenum phosphide is widely used as non-precious metal catalyst in the direction of hydrogen evolution. The introduction of carbon source can improve the conductivity and stability of the electrocatalyst. The introduction of nitrogen source can improve the hydrogen evolution performance in an alkaline solution.

## Testing the Hypothesis

### Materials

Urea (CH_4_N_2_O), glucose (C_6_H_12_O_6_), ammonium dihydrogen phosphate (NH_4_H_2_PO_4_), and ammonium heptamolybdate ((NH_4_)_6_Mo_7_O_24_·4H_2_O) were purchased from Sinopharm Chemical Reagent Co., Ltd. KOH was purchased from Aladdin Ltd. in Shanghai. Deionized water used in the experiment was from ultrapure water equipment.

### Sample Preparation

In the typical synthesis of MoP-NC, (NH4)_6_Mo_7_O_24_·4H_2_O (0.240 g), NH_4_H_2_PO_4_ (0.167 g), and CO(NH_2_)_2_ (2.000 g) were dissolved in 50 mL of deionized water and subjected to 15 min of sonication. Thereafter, the resulting solution was heated to 80 °C and magnetically stirred for 90 min, remained relatively closed throughout the reaction, and then was dried in a freeze dryer. The obtained white precursor powder was heated from room temperature to 900 °C at a rate of 5 °C/min under a N_2_ atmosphere for 120 min. In order to explore the influence of carbon source on material synthesis, MoP-C was prepared by using glucose instead of urea as carbon source. When no carbon source or phosphorus source was added, Bulk-MoP and Mo-NC were prepared, respectively.

### Characterizations

The X-ray diffraction (XRD) information was collected on an X-ray diffraction (XRD, Bruker D8-Advance diffractometer with Cu Kαradiation (*λ* = 1.54056 Å)). The microstructure of the sample was obtained by a field emission scanning electron microscopy (FE-SEM, S-4800, Hitachi, Japan). The TEM images were performed on a transmission electron microscopy (TEM, JEM-2100, JEOL, Japan). The chemical components were analyzed by an X-ray photoelectron spectroscopy (XPS) with Mg Kα as a monochromatic X-ray source.

### Electrochemical Test

All electrochemical measurements were performed on an electrochemical workstation (CHI 660E Chenhua, Shanghai) equipped with a conventional three-electrode system of Pine Modulated Speed Rotator (PINE, USA). The Pt wire and the saturated calomel electrode (SCE) corresponded to the counter electrode and the reference electrode, respectively, and the glassy carbon electrode and the rotating disk device were connected as a working electrode. In addition, 1 M KOH electrolyte was provided for testing. The working electrode was prepared as follows: First, 5 mg of the catalyst was dissolved in a solution mixed with 350 μL of isopropanol, 650 μL of deionized water, and 50 μL of 5 wt% Nafion. Next, after the abovementioned mixed solution was ultrasonicated for 30 min, a uniformly dispersed ink was obtained. Finally, 10 μL of the ink was dropped on a glassy carbon electrode (diameter, 5 mm) for natural air-drying treatment, wherein the catalyst had an areal density of 0.485 mg cm^−2^. In order to better describe the performance parameters of the sample, we used a Pt/C catalyst (20 wt%) for comparison, and the preparation process was the same as that of the above working electrode. A sweep speed of 10 mV s^−1^ was used for linear sweep voltammetry (LSV) measurements. Tafel was obtained by fitting the appropriate region curve according to the Tafel equation, and the electrochemical stability was obtained by performing 1000 cycles with the sweep speed of 100 mV·S^−1^. The double-layer capacitance (*C*_dl_) data is derived from cyclic voltammetry (CV), which is performed at the same voltage range (0.847–0.947 V vs RHE) at different scan rate ranges (20–200 mV). Electrochemical impedance spectroscopy (EIS) measurements were made at a constant potential amplitude of 10 mV over the default frequency range (1–10^5^ Hz).

## Implications of the Hypothesis

**Fig. 1:**
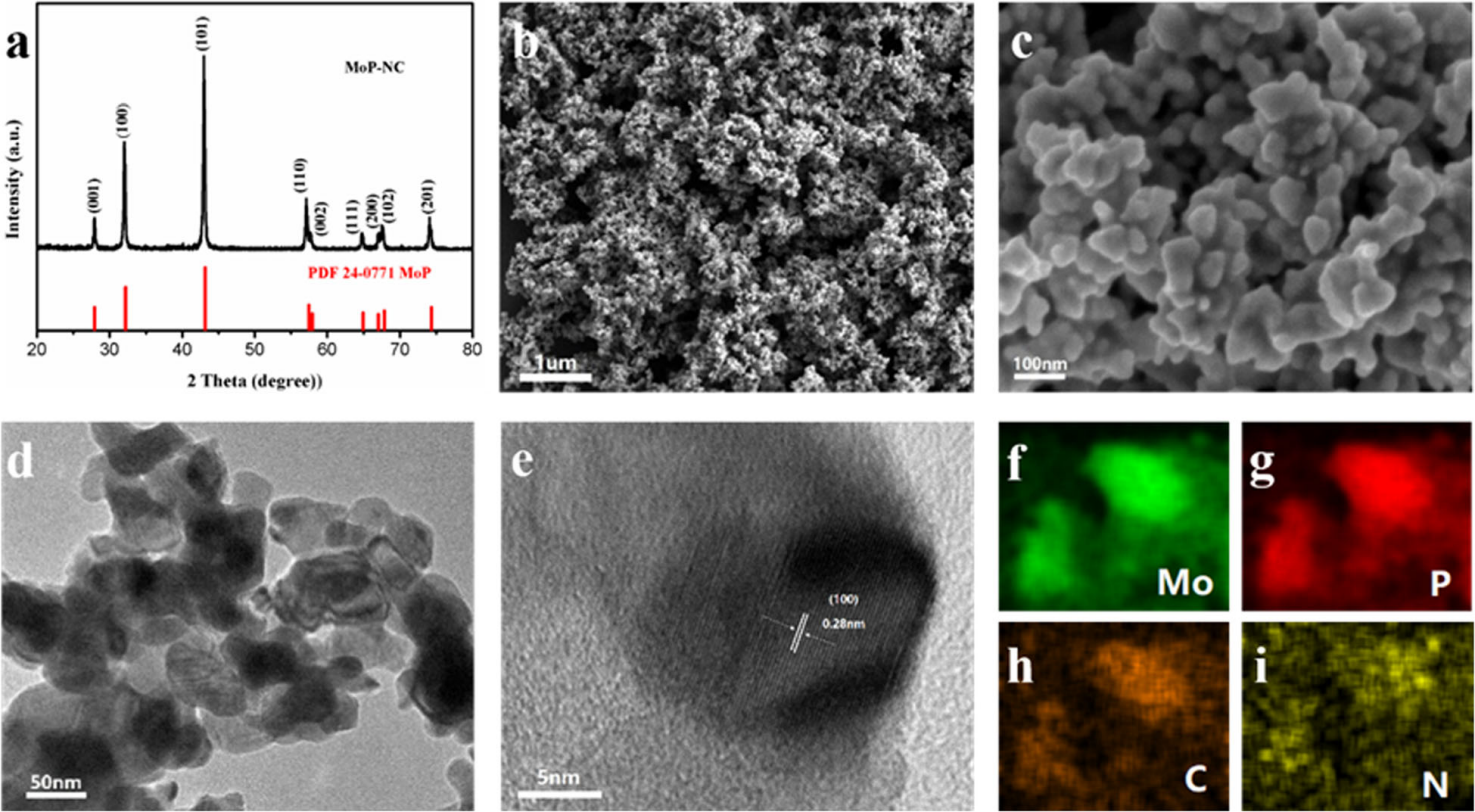
**a** XRD patterns for MoP. **b**, c SEM images of MoP-NC. **d**, **e** TEM and HRTEM images of MoP-NC. **f**–**i** corresponding EDS elemental mapping images of Mo, P, C, and N

Figure 1a showed the XRD pattern of MoP-NC presents diffraction peaks at 27.95, 32.17, 43.15, 57.48, 57.95, 64.93, 67.03, 67.86, and 74.33, corresponding to the nine different crystal faces of MoP. SEM images of MoP-NC showed amorphous small particles microstructures (Fig. 1b). The particles gathered together to form small clusters, but there was still a certain gap between the clusters. This small and dense structure made MoP-NC both have considerable catalytic activity and good stability (Fig. 1c). It can be seen from TEM and high-resolution TEM (HRTEM) (Fig. 1d, e) that these nanoparticles showed distinct lattice fringes, such as lattice fringes from the (100) plane with a pitch of 0.28 nm. In addition, outside these well-defined lattice fringe regions were MoP-NC nanoparticle edges, which strongly supported the incorporation of MoP-NC nanoparticles into the carbon matrix. The corresponding EDS element mapping image (Fig. 1f–i) further verified the uniform distribution of the four elements in the product MoP-NC.

**Fig. 2:**
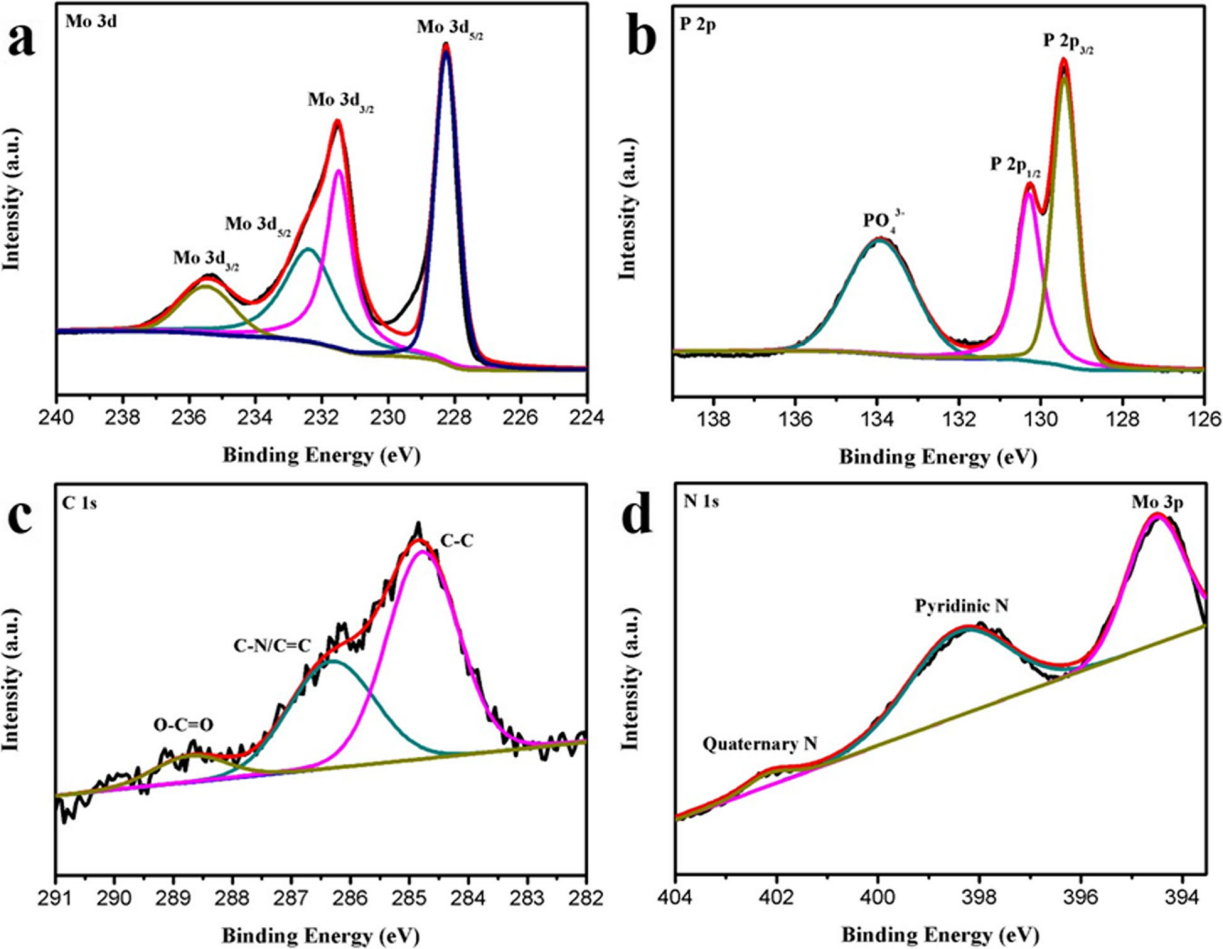
XPS spectra of (**a)** Mo 3d, (**b)** P 2p, (**c**) C 1s, and (**d**) N 1s regions

To further understand the elemental distribution of MoP-NC, XPS was characterized. In the Mo 3d spectral region, Mo contains two states of Mo^3+^ and Mo^6+^ (Fig. 2a). The presence of Mo 3d_3/2_ and Mo 3d_5/2_ in the Mo^3+^ state led to micro-vibration peaks at 231.5 and 228.2 ev, while the peaks of 235.5 and 232.4 ev were attributed to Mo 3d_3/2_ and Mo 3d_5/2_ in the Mo^6+^ state, because the surface of the MoP-NC material was inevitably oxidized in the air [24, 25]. In the P 2p region (Fig. 2b), micro-vibration peaks of 130.7 and 129.4 eV were assigned to P 2p_1/2_ and P 2p_3/2_, respectively, revealing the presence of P^3−^ [26]. The peak at 133.9 eV can be attributed to PO_4_^3−^ [27]. In the C 1s XPS spectrum (Fig. 2c), the main peaks corresponding to the three chemical bonds were 228.7 eV (O-C=O), 284.8 eV (C-N/C=C), and 286.3 eV (C-C), respectively [28]. The appearance of C-N/C=N suggested that some of the carbon atoms in MoP-NC were replaced by nitrogen atoms to form N-doped carbon. In the spectrum of N 1s (Fig. 2d), three different nitrogen environments can be solved from this region, wherein the peaks of 398.4 and 402.1 eV with larger binding energies corresponded to the major amounts of pyridinium and the minor amount of quaternary nitrogen, respectively. In addition, a peak at 394.5 eV was designated as a combination of N and Mo 3p [29].

**Fig. 3:**
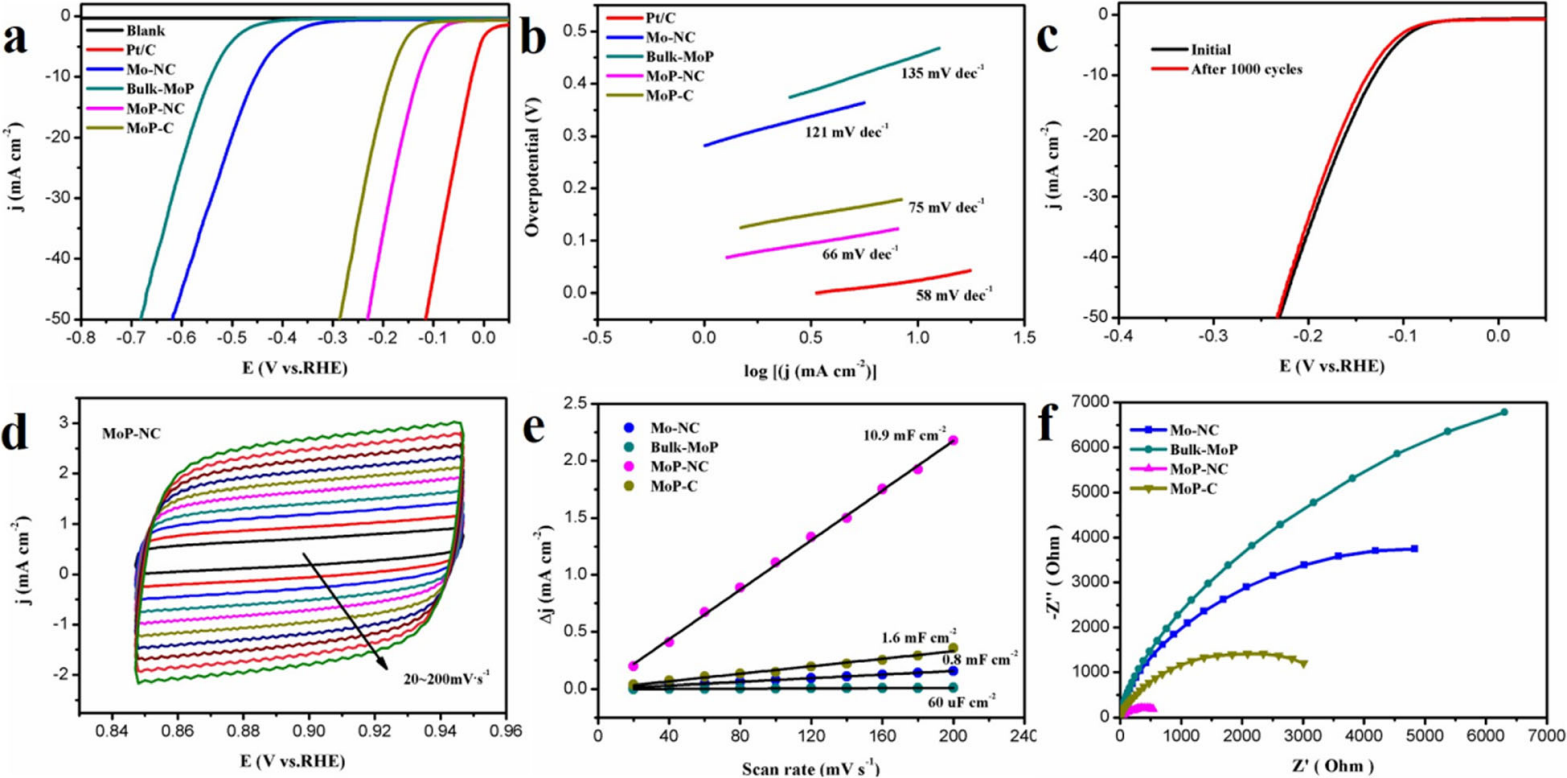
**a** LSV curves with scan rate of 10 mV s^−1^ at room temperature in 1 M KOH. **b** Tafel plots of the as-synthesized samples. **c** Stability of MoP-NC after 1000 cycles of voltammetry (CV) cycle. **d** CV plots of MoP-NC at a scan rate between 20 and 200 mV s^−1^. **e** Double-layer capacitor (*C*_dl_) of Mo-NC, Bulk-MoP, MoP-NC, and MoP-C with a capacitor current of 0.1 V. **f** The EIS spectra of Mo-NC, Bulk-MoP, MoP-NC, and MoP-C

The electrocatalytic HER activity of MoP-NC in 1 M KOH (pH = 14) used typical three-electrode system with the sweep speed of 10 mV s^−1^. Since the properties of the prepared materials required comparative analysis, Mo-NC, Bulk-MoP, and MoP-C were also studied. Figure 3a depicted LSV curves. The addition of Pt/C and a blank glassy carbon electrode (Blank) made the entire curves look more hierarchical. The overpotential of MoP-NC at a current density of 10 mA cm^−2^ requires only 131 mV, which was significantly better than that of Mo-NC and Bulk-MoP. Furthermore, the LSV performance of MoP-C prepared from glucose instead of urea as a carbon source was also dwarfed by MoP-NC. It is worth noting that compared to HER in an acidic solution, the rate of H ion production during the decomposition of HER water in the alkaline was slower (about 2–3 orders of magnitude lower than acidic activity) and had a larger challenging [30–32]. Figure 3b showed the fitted Tafel graph equation: *η* = *a* + *b* log *j*, where *b* is the Tafel slope and *j* is the current density [33]. The Tafel slope of Pt/C is 58 mV dec^−1^, compared with Mo-NC (121 mV dec^−1^), Bulk-MoP (135 mV dec^−1^), and MoP-C (75 mV dec^−1^), MoP-NC had a lower parameter of only 66 mV dec^−1^, indicating that the HER catalytic kinetics for the MoP-NC electrode were faster. At the same time, we found that the MoP-NC in this work was quite competitive with the HER performance of the previously reported Mo-based composite/carbon electrocatalyst (Table 1). As can be seen from Table 1, most of the MoP-based materials were based on acidic conditions and were rarely tested under alkaline conditions [17–19, 22–24, 34–37]. In addition, some of them had been tested in both acidic and alkaline environments [38–41]. However, only amorphous carbon-coated MoP materials in these materials performed better in alkaline environments than in acidic environments. The reason why MoP-NC in our work can achieve good HER performance in alkaline environment was because urea was used as both a carbon source and a nitrogen source in the synthesis process, during which it also decomposed some gas. It slowed down the polymerization of N and C co-doped MoP, which played an excellent auxiliary synthesis role. The stability of the prepared materials was continuous CV with a scan rate of 100 mV s^−1^. After 1000 cycles, the LSV curve had a small current density loss compared to the initial value (Fig. 3c). Figure 3d showed the CV plots of MoP-NC, which was performed at the same voltage range (0.847–0.947 V vs RHE) at different scan rate ranges (20–200 mV). To further explore the double-layer capacitance (*C*_dl_) of the material, the *C*_dl_ of a series of control groups was shown in Fig. 3e. The *C*_dl_ of Mo-NC, Bulk-MoP, and MoP-C were 0.8 mF cm^−2^, 60 μF cm^−2^ and 1.6 mF cm^−2^, respectively, while the *C*_dl_ of MoP-NC was 10.9 mF cm^−2^ which was much larger than the above materials. The suggestion indicated that MoP-NC had a higher active surface area. Furthermore, the conductivity of MoP-NC was evaluated by electrochemical impedance spectroscopy (EIS). Figure 3f showed the Nyquist diagram of the different catalysts. The charge transfer resistance of the MoP-NC catalyst was lower than that of other catalysts, which meant that the faster electron transfer ratio of the MoP-NC catalyst after N, C co-doping further enhanced the electrocatalytic performance of HER.

**Table 1:** Comparison of HER performance of MoP-NC with the reported Mo compounds/carbon electrocatalysts

Catalysts	Electrolyte	Catalyst loading (mg cm^−2^)	*η*_10_ (mV)	Tafel slope (mV dec^−1^)	Reference
MoP-NC	1 M KOH	0.485	131	66	This work
MoP@PC	0.5 M H_2_SO_4_	0.41	153	66	[16]
MoP networks	0.5 M H_2_SO_4_	0.36	125	54	[17]
MoP (nano)	0.5 M H_2_SO_4_	1	90	45	[18]
MoP-C	0.5 M H_2_SO_4_	0.84	169	82	[21]
MoP@NC	0.5 M H_2_SO_4_	0.70	135	57	[22]
MoP	0.5 M H_2_SO_4_	0.86	137	54	[23]
MoP sheets	0.5 M H_2_SO_4_	0.357	172	54.5	[31]
Mo2C/MoP@NPC	0.5 M H_2_SO_4_	/	160	75	[32]
MoP@NC-CF	0.5 M H_2_SO_4_	0.36	125	53	[33]
CQDs/MoP	1 M KOH	/	172	56	[34]
MoP/NC	0.5 M H_2_SO_4_	0.56	120	52	[35]
	1 M KOH		170	50	
MoP-RGO	0.5 M H_2_SO_4_	0.42	117	62	[36]
	1 M KOH		150	62	
MoP/Mo_2_C@C	0.5 M H_2_SO_4_	0.453	89	45	[37]
	1 M KOH		75	58	
N-MoP@C	0.5 M H_2_SO_4_	0.509	150	62	[38]
	1 M KOH		125	49	

## Conclusions

In summary, we synthesized the amorphous small particles of MoP-NC by a simple and efficient two-step method. Since the MoP nanoparticles were coated with carbon, they partially aggregated together. Fortunately, this structure does not drag the performance of the material itself but also contributes to its stability. This overall dispersion, locally aggregated small-particle material reached a current density of 10 mA cm^−2^ in 1 M KOH requiring only an overpotential of 131 mV, which is superior to the reported HER performance of a single molybdenum phosphide material in an alkaline environment. In addition, the material exhibited negligible performance degradation even after scanning 1000 CV cycles. Our results show that carbon-coated MoP can also overcome the alkaline environment to achieve excellent HER electrocatalytic activity and stability.

## Data Availability

All data are fully available without restriction.

## Abbreviations

CA: Citric acid
*C*_dl_: The double-layer capacitance
CV: Cyclic voltammetry
EIS: Electrochemical impedance spectroscopy
HER: Hydrogen evolution reaction
HRTEM: High-resolution transition electronmicroscopy
KOH: Potassium hydroxide
LSV: Linear sweep voltammetry
MoP: Molybdenum phosphide
MoP-NC: N, C co-doped MoP
SCE: Saturated calomel electrode
SEM: Scanning electron microscopy
TEM: Transition electronmicroscopy
TMPs: Transition metal phosphides
TOP: Trioctylphosphine
XPS: X-ray photoelectron spectroscopy
XRD: X-ray powder diffraction

## References

[1] Yuan W, Feng Y, Xie A (2016). Nitrogen-doped nanoporous carbon derived from waste pomelo peel as a metal-free electrocatalyst for the oxygen reduction reaction. Nanoscale.

[2] Jiao Y, Zheng Y, Jaroniec M (2015). Design of electrocatalysts for oxygen-and hydrogen-involving energy conversion reactions. Chem.Soc.Rew..

[3] Chakrabartty S, Raj CR (2018). Mo_2_C@ NC nanowire bundle for efficient electrocatalytic hydrogen evolution. Int. J. Hydrogen Energ..

[4] Michalsky R, Zhang YJ, Peterson AA (2014). Trends in the hydrogen evolution activity of metal carbide catalysts. ACS Catal..

[5] Kumar R, Rai R, Gautam S (2017). Nano-structured hybrid molybdenum carbides/nitrides generated in situ for HER applications. J. Mater. Chem. A.

[6] Nørskov JK, Bligaard T, Logadottir A (2005). Trends in the exchange current for hydrogen evolution. J. Electrochem.l Soc.

[7] Pan Y, Liu Y, Liu C (2015). Phase-and morphology-controlled synthesis of cobalt sulfide nanocrystals and comparison of their catalytic activities for hydrogen evolution. Appl. Surf. Sci..

[8] Merki D, Hu X (2011). Recent developments of molybdenum and tungsten sulfides as hydrogen evolution catalysts. Energ. Environ. Sci..

[9] Wang L, Liu X, Luo J (2017). Self-optimization of the active site of molybdenum disulfide by an irreversible phase transition during photocatalytic hydrogen evolution. Angew. Chem..

[10] Ito Y, Cong W, Fujita T (2015). High catalytic activity of nitrogen and sulfur co-doped nanoporous graphene in the hydrogen evolution reaction. Angew. Chem. Int. Edit..

[11] Xiong J, Li J, Shi J (2018). In situ engineering of double-phase interface in Mo/Mo_2_C heteronanosheets for boosted hydrogen evolution reaction. ACS Energy Lett..

[12] Pi M, Zhang D, Wang S (2018). Enhancing electrocatalytic hydrogen evolution of WP2 three-dimensional nanowire arrays via Mo doping. Materials Letters.

[13] Zhang Y, Li P, Yang X (2018). High-efficiency and stable alloyed nickel based electrodes for hydrogen evolution by seawater splitting. J. Alloy. Compd..

[14] Yue Q, Wan Y, Sun Z (2015). MoP is a novel, noble-metal-free cocatalyst for enhanced photocatalytic hydrogen production from water under visible light[J]. J. Mater. Chem. A.

[15] Liang X, Zhang D, Wu Z (2016). The Fe-promoted MoP catalyst with high activity for water splitting. Appl. Catal. A-Gen..

[16] Anjum MAR, Lee JS (2017). Sulfur and nitrogen dual-doped molybdenum phosphide nanocrystallites as an active and stable hydrogen evolution reaction electrocatalyst in acidic and alkaline media. ACS Catal..

[17] Yang J, Zhang F, Wang X (2016). Porous molybdenum phosphide nano-octahedrons derived from confined phosphorization in UIO-66 for efficient hydrogen evolution. Angew. Chem. Int. Edit..

[18] Xing Z, Liu Q, Asiri AM (2014). Closely interconnected network of molybdenum phosphide nanoparticles: a highly efficient electrocatalyst for generating hydrogen from water. Adv. mater..

[19] McEnaney JM, Crompton JC, Callejas JF (2014). Amorphous molybdenum phosphide nanoparticles for electrocatalytic hydrogen evolution. Chem.Mater..

[20] Wang Y, Nie Y, Ding W (2015). Unification of catalytic oxygen reduction and hydrogen evolution reactions: highly dispersive Co nanoparticles encapsulated inside Co and nitrogen co-doped carbon. Chem. Commun..

[21] Cui W, Liu Q, Xing Z (2015). MoP nanosheets supported on biomass-derived carbon flake: One-step facile preparation and application as a novel high-active electrocatalyst toward hydrogen evolution reaction. Appl. Catal. B-Environ..

[22] Wu Z, Wang J, Liu R (2017). Facile preparation of carbon sphere supported molybdenum compounds (P, C and S) as hydrogen evolution electrocatalysts in acid and alkaline electrolytes. Nano Energy.

[23] Gao S, Liu Y, Li GD (2016). General urea-assisted synthesis of carbon-coated metal phosphide nanoparticles for efficient hydrogen evolution electrocatalysis. Electrochim. Acta.

[24] Xiao P, Sk MA, Thia L (2014). Molybdenum phosphide as an efficient electrocatalyst for the hydrogen evolution reaction. Energ. Environ. Sci..

[25] Wang T, Du K, Liu W (2015). Enhanced electrocatalytic activity of MoP microparticles for hydrogen evolution by grinding and electrochemical activation. J. Mater. Chem. A.

[26] Zhang X, Liu Y, Xiong Q (2017). Vapour-phase hydrothermal synthesis of Ni_2_P nanocrystallines on carbon fiber cloth for high-efficiency H2 production and simultaneous urea decomposition. Electrochim. Acta.

[27] Ojha K, Sharma M, Kolev H (2017). Reduced graphene oxide and MoP composite as highly efficient and durable electrocatalyst for hydrogen evolution in both acidic and alkaline media. Catal. Sci. Technol..

[28] Pu Z, Amiinu IS, Liu X (2016). Ultrastable nitrogen-doped carbon encapsulating molybdenum phosphide nanoparticles as highly efficient electrocatalyst for hydrogen generation. Nanoscale.

[29] Yan H, Xie Y, Jiao Y (2018). Holey reduced graphene oxide coupled with an Mo_2_N–Mo_2_C heterojunction for efficient hydrogen evolution. Adv. Mater..

[30] Wang L, Liu X, Zhang Q (2019). Quasi-one-dimensional Mo chains for efficient hydrogen evolution reaction. Nano Energy.

[31] Wang S, Zhang L, Xia Z (2012). BCN graphene as efficient metal-free electrocatalyst for the oxygen reduction reaction. Angew. Chem. Int. Edit..

[32] Zheng Y, Jiao Y, Vasileff A (2018). The hydrogen evolution reaction in alkaline solution: from theory, single crystal models, to practical electrocatalysts. Angew. Chem. Int. Edit..

[33] Yan D, Li F, Xu Y (2018). Three-dimensional reduced graphene oxide–Mn_3_O_4_ nanosheet hybrid decorated with palladium nanoparticles for highly efficient hydrogen evolution. Int. J. Hydrogen Energy.

[34] Jia J, Zhou W, Li G (2017). Regulated synthesis of Mo sheets and their derivative MoX sheets (X: P, S, or C) as efficient electrocatalysts for hydrogen evolution reactions. ACS appl. Mater. inter..

[35] Chi JQ, Gao WK, Lin JH (2018). Nitrogen, phosphorus dual-doped molybdenum-carbide/molybdenum-phosphide-@-carbon nanospheres for efficient hydrogen evolution over the whole pH range. J. colloid interf. Sci..

[36] Guo Z, Liu P, Liu J (2018). Neural network inspired design of highly active and durable N-doped carbon interconnected molybdenum phosphide for hydrogen evolution reaction. ACS Appl. Energy Mater..

[37] Zhang L, Yang Y, Ziaee MA (2018). Nanohybrid of carbon quantum dots/molybdenum phosphide nanoparticle for efficient electrochemical hydrogen evolution in alkaline medium. ACS appl. Mater. Inter..

[38] Huang Y, Song X, Deng J (2019). Ultra-dispersed molybdenum phosphide and phosphosulfide nanoparticles on hierarchical carbonaceous scaffolds for hydrogen evolution electrocatalysis. Appl. Catal. B-Environ..

[39] Wu Z, Wang J, Zhu J (2017). Highly efficient and stable MoP-RGO nanoparticles as electrocatalysts for hydrogen evolution. Electrochim. Acta.

[40] Zhang LN, Li SH, Tan HQ (2017). MoP/Mo_2_C@ C: a new combination of electrocatalysts for highly efficient hydrogen evolution over the entire pH range. ACS Appl. Mater. Inter.

[41] Liu X, Zhang L, Li M (2018). Tandem MoP nanocrystals with rich grain boundaries for efficient electrocatalytic hydrogen evolution. Chem. Commun.

